# Failure to CAPTCHA Attention: Null Results from an Honesty Priming Experiment in Guatemala

**DOI:** 10.3390/bs7020028

**Published:** 2017-04-28

**Authors:** Stewart Kettle, Marco Hernandez, Michael Sanders, Oliver Hauser, Simon Ruda

**Affiliations:** 1 Behavioural Insights Team, 4 Matthew Parker St, Westminster, London SW1H 9NP, UK; stewart.kettle@bi.team (S.K.); simon.ruda@bi.team (S.R.); 2 World Bank, Praterstrasse 31, 21 stock, Vienna 1020, Austria; marcohernandez@worldbank.org; 3 Blavatnik School of Government, Oxford University, 120 Walton St, Oxford OX2 6GG, UK; 4 Harvard Business School, Boston, MA 02163, USA; ohauser@hbs.edu; 5 Harvard Behavioral Insights Group, Harvard Kennedy School, Taubman Building, First Floor, 15 Eliot Street, Cambridge, MA 02138, USA

**Keywords:** tax compliance, behavioural economics, randomised field experiments, international development

## Abstract

We report results from a large online randomised tax experiment in Guatemala. The trial involves short messages and choices presented to taxpayers as part of a CAPTCHA pop-up window immediately before they file a tax return, with the aim of priming honest declarations. In total our sample includes 627,242 taxpayers and 3,232,430 tax declarations made over four months. Treatments include: honesty declaration; information about public goods; information about penalties for dishonesty, questions allowing a taxpayer to choose which public good they think tax money should be spent on; or questions allowing a taxpayer to state a view on the penalty for not declaring honestly. We find no impact of any of these treatments on the average amount of tax declared. We discuss potential causes for this null effect and implications for ‘online nudges’ around honesty priming.

## 1. Introduction

Improving the fairness, transparency, efficiency and effectiveness of tax systems will be critical to achieving the sustainable development goals [1]. Where countries such as Guatemala fail to collect tax revenue effectively from their citizens, there are consequences for the ability to deliver public services and, where tax compliance is patchy, general confidence in the government’s competence and fairness may also be compromised. This natural field experiment involved priming honesty among Guatemalan taxpayers filing online declarations for income tax or value-added tax (VAT). Treatment messages were included as part of a pop-up CAPTCHA (Completely Automated Public Turing test to tell Computers and Humans Apart) window on the tax declaration website (Declaraguate) immediately prior to individuals reaching a declaration form. The large-scale trial involves all people who completed an online tax return between August 2014 and January 2015 (627,242 individuals, completing a total of 3.2 million tax returns).

Randomised controlled trials in a natural context, commonly referred to as natural field experiments in the social sciences [2,3], are becoming the gold standard for evidence-based policy-making, and their increased use is being advocated in governments and organisations [4,5,6,7]. Field experiments encouraging honest tax reporting, in particular, have a long tradition among economists, psychologists and policy scholars [8,9,10,11,12]. Some have found that the mention of increased audits or penalties has a positive impact on tax compliance, e.g., [13,14,15]. These interventions are in part based on laboratory or survey experiments that have studied what structural factors promote contributions to public goods in general (e.g., [7,16,17]) and paying taxes in particular (e.g., [18,19]). Others have been able to prompt more compliance using behaviourally inspired messages (often referred to as ‘nudges’, [20]. For example, Hallsworth et al. [21] found in a large-scale field experiment in the United Kingdom that letters communicating the widespread social norm of paying taxes on time increases the fraction of taxpayers who pay their taxes on time. In a similar experiment in Guatemala, Kettle et al. [12] showed that behaviourally informed letters more than triple receipts from late income tax filers. Perez-Truglia and Troiano [22] demonstrated in a field experiment that a ‘shaming’ policy can be effective in reducing tax delinquency.

Taxpayers in our trial were randomly allocated to either see one of six messages, or be part of a control group, which saw the original CAPTCHA without any behavioural message. The trial finds no differential impact between the original CAPTCHA (which contains no behavioural prompts) and various prompts inspired by behavioural science. Treatments included an honesty declaration, information about public goods paid for by taxes and punishment for noncompliance, and treatments that allowed participants to choose what they believe to be a good use of public funds, or an appropriate punishment. The results show that none of our treatments had a significant impact on tax declarations in this context. The fact that all of the six treatments were found to be ineffective (rather than some) supports the hypothesis that the setting in which the information was conveyed may have been crucial here, rather than the content of the messages.

## 2. Materials and Methods

### 2.1. Tax Regime Selection

The trial involves Guatemalan taxpayers making declarations for income tax and value added tax (VAT), of which there are two regimes for each. For income tax the default option is a gross income tax, which entails a six percent direct tax on gross revenue. This option is also associated with simplified accounting standards. The alternative is for taxpayers to self-select into a profits tax at the beginning of the year, which entails a tax rate of 28 percent (in 2014) on taxable income or profit.

The two regimes for VAT both entail a 12 percent tax on the value of products. They differ, however, in terms of eligibility and payment procedures. Taxpayers with an annual turnover of more than GTQ 150,000 (approx. $19,200 USD at the time of the experiment) are part of the general regime, while other taxpayers are part of the small taxpayers’ regime. VAT is charged on sales of goods within the country, sale of services in the country, any imported goods, leasing contracts, transfers of real estate, and insurance and bond sales. Exports, banking activities, payments in-kind, mergers, trade in financial instruments, and trust arrangements are exempt from VAT. We consider taxpayers under both regimes.

The trial involves all Guatemalans who declare their tax online. For income tax and VAT general, online declaration is mandatory, while for VAT small taxpayers over 50 percent of declarations are made online. However, in conversations with government officials, we learned that a large fraction of Guatemalans are likely to be unregistered and thus not captured by the tax system. Our interventions will thus only affect the fraction of citizens who have already made an ethical decision to register for tax filing, although we note that this limitation is common to all tax compliance field experiments.

There are reasons to expect different treatment effects depending on the tax being declared. The profits regime is charged at a higher rate than the simplified regime and so the incentive to behave dishonestly may be higher (for a more thorough discussion of this, see Kettle et al. [12]). Individuals and businesses that complete this kind of return may, however, be more affluent, which in turn could reduce their incentive to be dishonest due to a lower marginal value of money, consistent with the model put forward by Allingham and Sandmo [23]. The simplified option is, as its name implies, a less complex form. This may lead to greater honesty as there are fewer dimensions across which it is possible to obfuscate tax liability. Additionally, the differences in timing for declarations may affect behaviour. For VAT small taxpayers, and the gross income tax regime, declarations are made monthly. These smaller and more frequent declarations may create more of an incentive to behave dishonestly as the declarations are so common, or less so as the payments are smaller.

### 2.2. Sample Selection and Random Treatment Assignment

Our initial sample involves the universe of taxpayers that made a declaration for any of the four tax regimes outlined above between August 2014 and January 2015. There are no restrictions to eligibility; however, due to missing observations for some variables (and in particular for the lagged value of declarations), our sample size for analysis is reduced from 715,190 participants to 627,242 participants. In total the trial involves 3,232,430 declarations by these 627,242 individuals. Table 1 shows the random assignment of individuals across the seven trial arms as well as summary statistics on demographics.

Randomisation is conducted at the individual level with no stratification. Randomisation was built into www.declaraguate.gt, the website for tax declaration owned by the Guatemalan Tax Authority (SAT), using a JavaScript random number generator that randomly draws an integer between 1 and 7 inclusive, using a standard (publicly available) algorithm. When an individual begins their tax return this provides an instruction to the declaration website to display one of the seven treatment arms. Randomisation is recorded by SAT as a field in the tax return data.

Individual randomisation was chosen as it is the most statistically efficient mechanism for randomisation, in terms of both analysis and the quality of randomisation. In practice, this means that individuals submitting multiple returns within the trial period are treated by a randomly assigned treatment for each declaration. Contamination between individuals was not believed to be a significant enough issue to warrant clustering the randomisation at a level above the individual. As individuals are identified after treatment, individuals are assigned afresh on each individual visit. We discuss the effects of first-time exposure to any condition and the repeated exposure to multiple conditions separately.

### 2.3. Experimental Design and Treatments

Taxpayers are randomly assigned to one of seven arms; a control arm where taxpayers receive the original CAPTCHA from the Declaraguate website, and six adapted versions that entail viewing or interacting with a behavioural prompt in addition to typing the original CAPTCHA. The CAPTCHA appears after the taxpayer selects the tax form to fill in on the main Declaraguate website, and before the form page. Table 2 below presents an overview of the seven treatment arms which we then describe in further detail below. The original Spanish versions of the treatment CAPTCHAs (and translations) are included in Appendix A. The ‘customer journey’ of taxpayers through the websiteis included in Appendix B (Figure A7, Figure A8, Figure A9, i.e., the website and forms between which the CAPTCHAs appear).

The treatment arms are placed directly below where the characters are typed for the CAPTCHA, but above the ‘fill form’ button which the taxpayer must press to continue to the next form. Some of the treatment CAPTCHAs involve specific actions that the taxpayer has to complete before they are able to press the ‘fill form’ button. These are described further below.

#### 2.3.1. Control Group

This CAPTCHA is the original used in the Declaraguate website. On the website the CAPTCHA appears as a pop up box. The text states ‘please type the characters that you see in the picture’. Below this is a picture containing jumbled but legible characters which need to be typed out by the taxpayer. Once the characters from the picture are typed, the taxpayer is then able to click on the ‘fill form’ button to take them to the declaration form.

#### 2.3.2. Honesty Declaration

**Figure A1:**
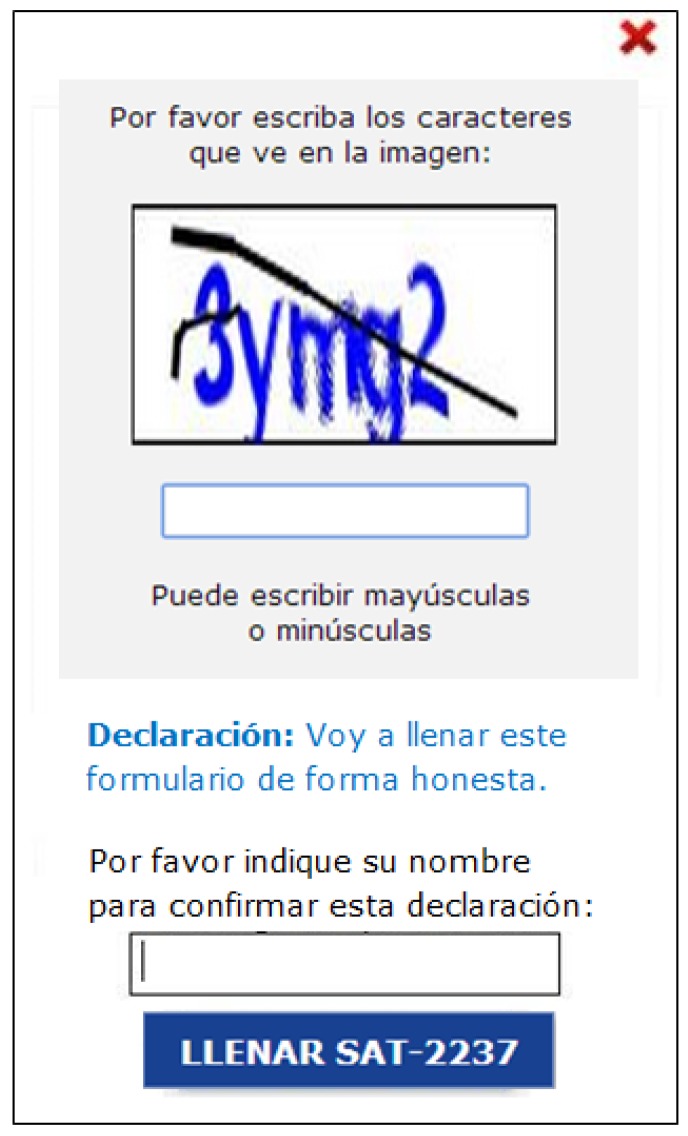
(T1) Honesty Declaration. Translation for text across all CAPTCHAs: ‘Please type the characters that you see in the picture’, and ‘You can type upper or lower case’. Treatment translation: ‘Declaration: I will fill out this form honestly, Please sign your name to confirm this declaration’. (The taxpayer must then enter their name in a box below this statement before being able to press the ‘fill form’ button.)

This CAPTCHA includes an honesty declaration that translates as: ‘Declaration: I will fill out this form honestly. Please sign your name to confirm this declaration’. The taxpayer must then enter their name in a box below this statement before being able to press the ‘fill form’ button. The treatment can be seen in Figure A1.

The Honesty Declaration CAPTCHA is based around the idea that most people see themselves as honest and act according to the ethical codes of society, but need reminders that these ethical codes should apply to all their decision-making. For instance, research has found that people are more honest when they are asked to recall the Ten Commandments just before making decisions where they have an option to be dishonest [24].

Specifically in the context of filing forms, Shu et al. [25] show that moving an honesty declaration to the start of a car insurance form increases truthful reporting of mileage driven each year. The intervention was simple but very effective: while traditionally people would fill out their annual insurance form and then sign their name at the bottom of the form, researchers moved both the honesty declaration and signature box to the top of the insurance form before they would fill it out. The intervention increased the number of miles declared by 10%. Other studies, for example Bhanot [26], who investigated the effect of pledges on loan repayment rates, found somewhat smaller effects, suggesting that this effect may have limitations.

The ‘honesty declaration CAPTCHA’ applies the same considerations to tax declarations. We hypothesise that when participants sign their full name below an honest declaration, their moral code is made more salient and hence they will be more likely to comply with it.

#### 2.3.3. Public Good

**Figure A2:**
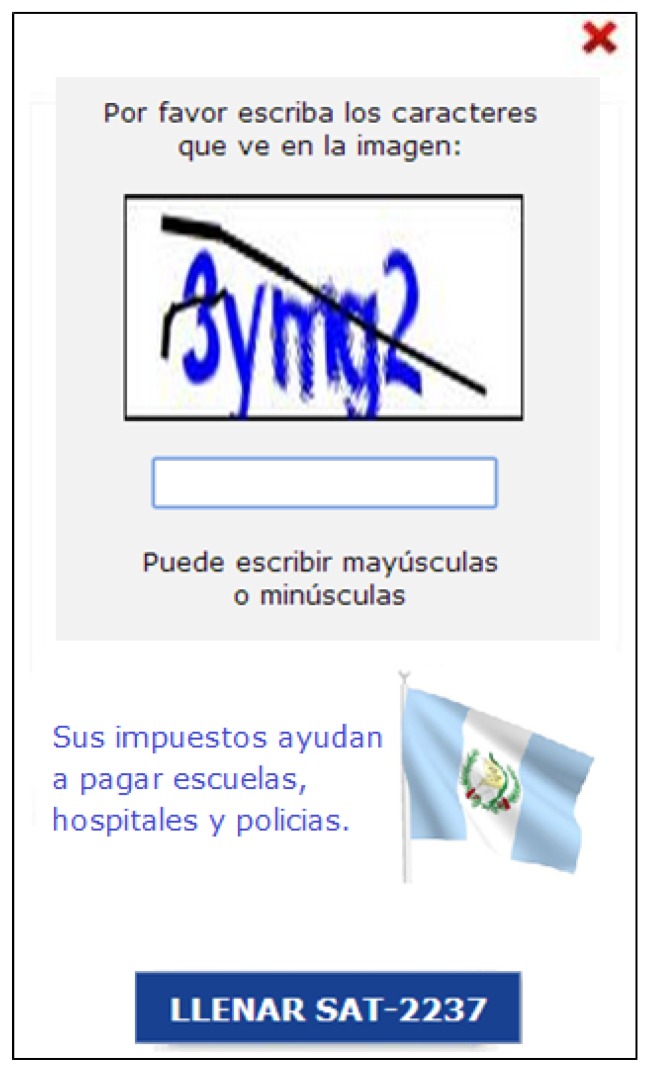
(T2) Public Good. Translation for text across all CAPTCHAs: ‘Please type the characters that you see in the picture’, and ‘You can type upper or lower case’. Treatment translation: ‘In 2013 your taxes helped pay for schools, hospitals and policemen.’

This CAPTCHA involves an image of the Guatemalan flag and the following public good message: ‘In 2013 your taxes helped pay for schools, hospitals and policemen.’ The treatment can be seen in Figure A2.

The Public Good CAPTCHA draws on the ‘non-deterrence’ approach to taxation; that citizens are fundamentally predisposed to cooperate with tax authorities. The approach contends that taxpayers’ decisions are not based on utility maximisation alone, but are influenced by morality, social norms and public goods, amongst other concepts [11,27,28,29]. In this view, the relationship between tax authority and taxpayer is basically cooperative, with the latter asking ‘what should I do?’ rather than ‘what can I get away with?’ [30]. The approach we take here is similar to Hallsworth [17].

In terms of administrative policy, the non-deterrence approach recommends that taxpayers are treated fairly and respectfully. They should be provided with clear and helpful information in order to make decisions. The authority should create a helpful service that makes it easy to comply. The route to reducing non-compliance is to persuade taxpayers by emphasizing that tax compliance is an ethical activity that is practised by the great majority of people, and that it creates valued public goods [28]. Following from this, the CAPTCHA is designed to prime taxpayers to think about the public goods that tax money is spent on. We hypothesise that people who consider the benefits of their own contribution will weigh this more heavily, relative to their own private consumption than they would do otherwise, and so will be more likely to make an honest declaration.

#### 2.3.4. Enforcement

**Figure A3:**
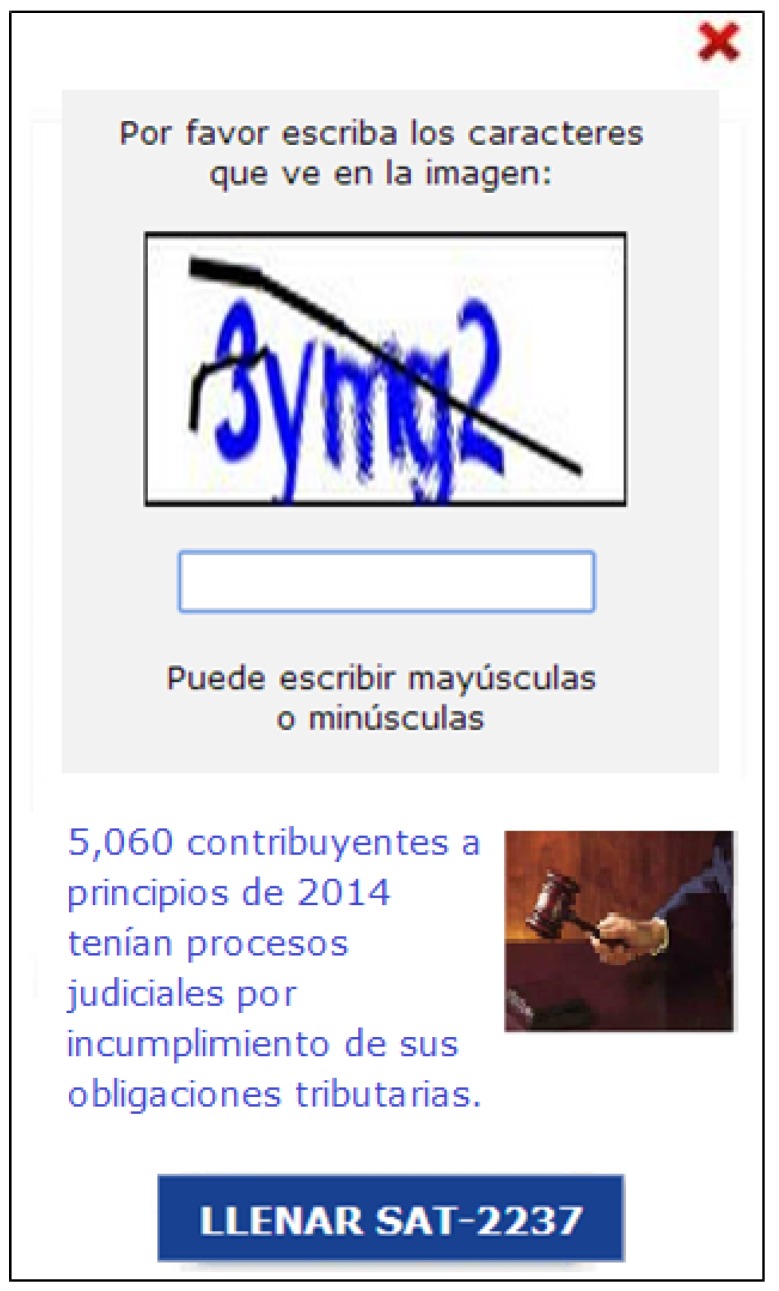
(T3) Enforcement. Translation for text across all CAPTCHAs: ‘Please type the characters that you see in the picture’, and ‘You can type upper or lower case.’ Treatment translation: ‘5060 taxpayers in early 2014 had legal proceedings for breach of their tax obligations.’

This CAPTCHA involves an image of a gavel and the following text: ‘5060 taxpayers in early 2014 had legal proceedings for breach of their tax obligations.’ The treatment can be seen in Figure A3.

The Enforcement CAPTCHA is based on the idea that signals of punishment enforcement can be a powerful deterrence against unlawful actions [31]. Knowledge that others have been caught for a crime can lead people to overestimate the likelihood that they could be the next to be caught [32].

Enforcement messages to increase taxation are based on the original economic model used to analyse tax compliance developed by Allingham and Sandmo [23]. The model sees taxpayers as rational utility maximisers and suggests that a taxpayer’s decision of whether to pay or evade tax is based on the trade-off between the monetary cost of complying and the expected cost of evading. The expected cost of evading is in turn based on the probability of getting caught, the probability of punishment if caught, and the punishment for which they would be liable. Accordingly, this ‘deterrence’ model predicts that the way to deter tax evasion is through increased sanctions for noncompliance.

The enforcement CAPTCHA draws on making enforcement more salient to the taxpayer. Similarly to the above, our hypothesis is that information on potential costs makes these costs more salient and so adjusts relative weights of private consumption and taxpaying for the participant. Enforcement messages on tax letters have been shown to be effective in a number of countries including Argentina and Venezuela [33,34].

#### 2.3.5. Choice Public Good

**Figure A4:**
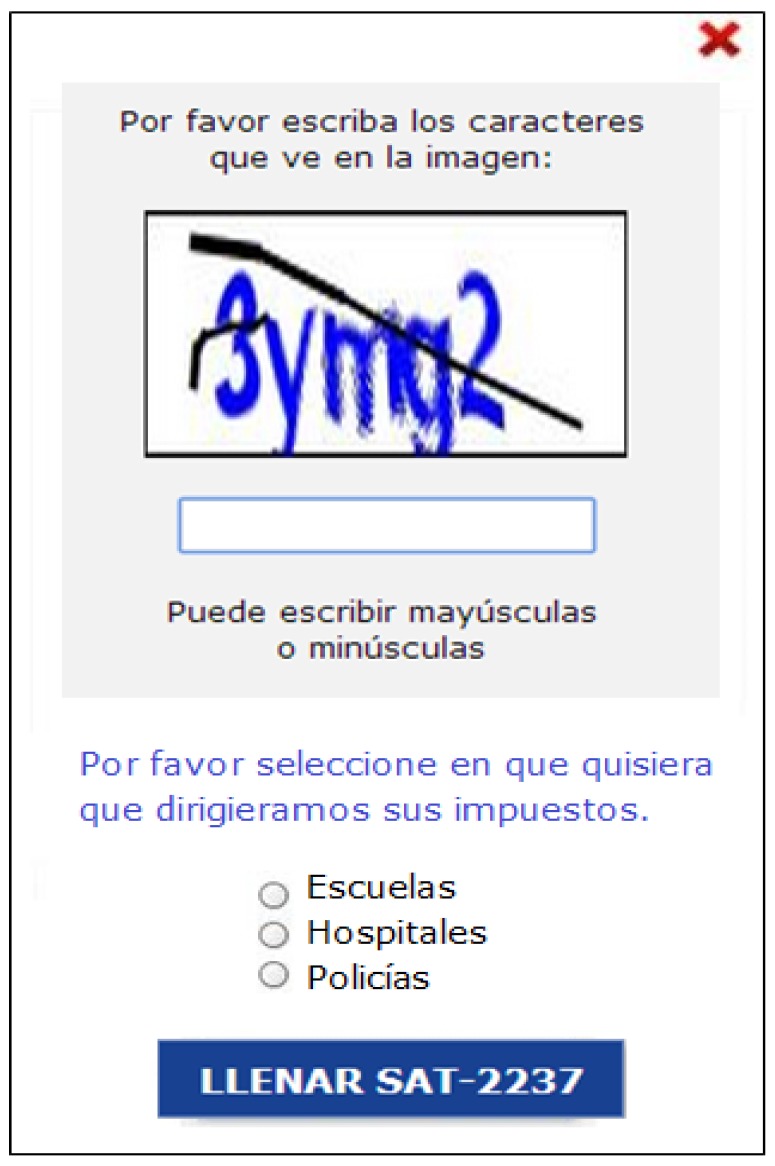
(T4) Choice Public Good. Translation for text across all CAPTCHAs: ‘Please type the characters that you see in the picture’, and ‘You can type upper or lower case.’ Treatment translation: ‘Please choose what you want us to direct your tax money to:’ The taxpayer is then given the choice of selecting ‘schools’, ‘hospitals’, or ‘policemen’.

This CAPTCHA gives the taxpayer a choice of public goods that they would like to see tax money spent on. The text of the CAPTCHA reads: ‘Please choose what you want us to direct your tax money to:’ The taxpayer is then given the choice of selecting ‘schools’, ‘hospitals’, or ‘policemen’. The treatment can be seen in Figure A4.

The design of this CAPTCHA incorporates a choice design with a public goods message. Research shows that people like to have a say in the design of a product or policy [35]. It can thus be effective to allow people to give an opinion on the cause towards which their tax money should go. This hypothesis is supported by Lamberton, De Neve & Norton [36] who find that participants who have had a chance to express non-binding preferences are 16% more likely to comply with tax instituted in a laboratory setting.

The intervention aims to prime the taxpayer into thinking about the public goods that tax goes towards, giving them the option to voice their preference. Our hypothesis is that even a cursory sense of control over public spending provides a sense of ownership and hence leads to taxpaying being more highly valued. The number of choices is limited to three in order to avoid potential cognitive exhaustion before filling out the form [37].

#### 2.3.6. Choice Enforcement

**Figure A5:**
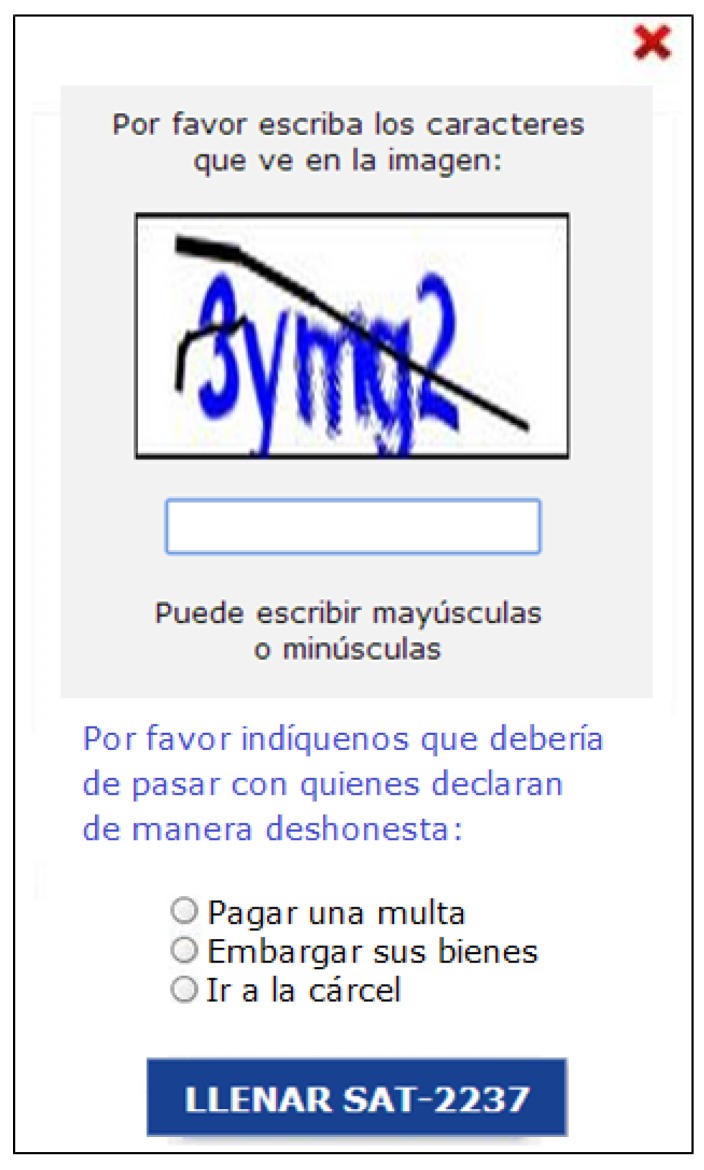
(T5) Choice Enforcement. Translation for text across all CAPTCHAs: ‘Please type the characters that you see in the picture’, and ‘You can type upper or lower case.’ Treatment translation: ‘Please tell us what you think should happen to people who fill out their forms dishonestly’. The taxpayer is then given the choice of selecting; ‘pay a fine’, ‘confiscate your assets’, or ‘go to jail’.

This CAPTCHA gives the taxpayer a choice of the punishment that they think people should receive for fraudulently declaring their tax: ‘Please tell us what you think should happen to people who fill out their forms dishonestly’. The taxpayer is then given the choice of selecting; pay a fine, confiscate your assets, or go to jail. The treatment can be seen in Figure A5.

This design is based on the same premise as above but incorporates punishment choices rather than public good choices. This CAPTCHA aims to prime the taxpayer into thinking about what should happen to people who do not pay their tax honestly.

The message also aims to invoke cognitive dissonance [38] in taxpayers who would have filled out the form dishonestly. We hypothesise that participants will suffer from cognitive dissonance due to the clash between their beliefs that dishonest declarers should be punished, and their dishonest actions. Under this hypothesis, participants would seek to resolve this dissonance by bringing their actions in line with their beliefs.

#### 2.3.7. Self-Select ‘I Am Honest’

**Figure A6:**
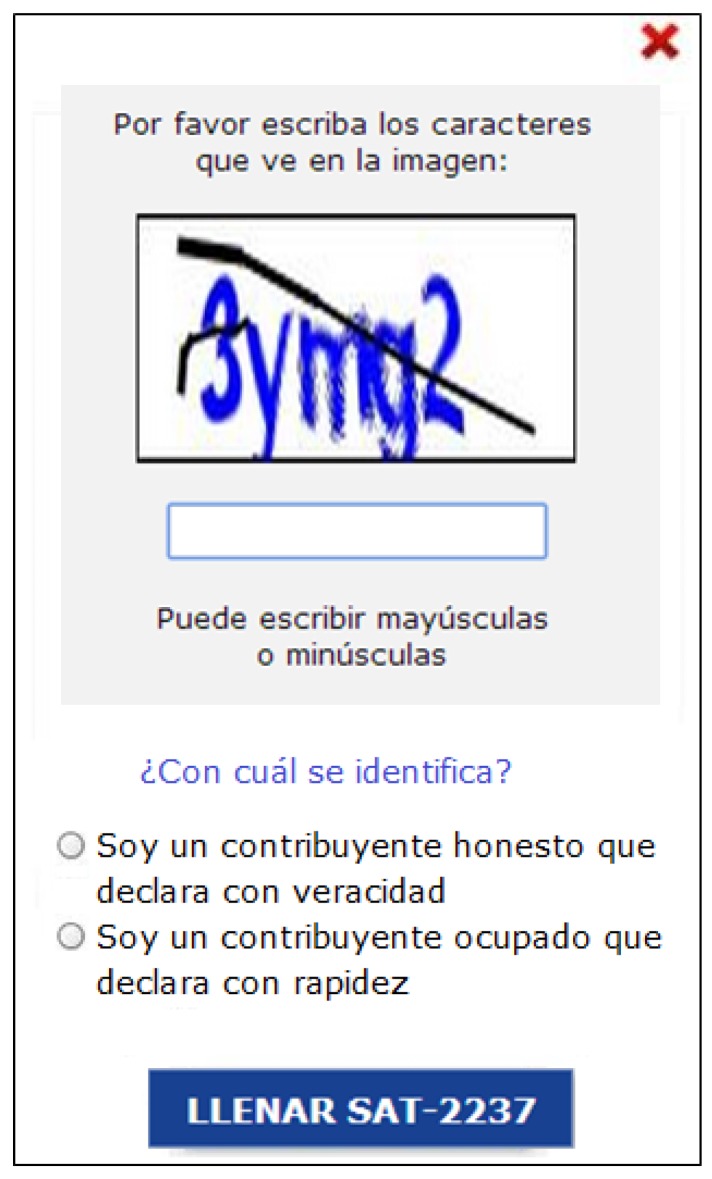
(T6) Selection ‘I am and honest taxpayer’. Translation for text across all CAPTCHAs: ‘Please type the characters that you see in the picture’, and ‘You can type upper or lower case’. Treatment translation: ‘Which of the following do you identify with?’ The taxpayer then has to select one of the following two options; ‘I am an honest taxpayer who declares truthfully’ or ‘I am a busy taxpayer who declares quickly’.

**Figure A7:**
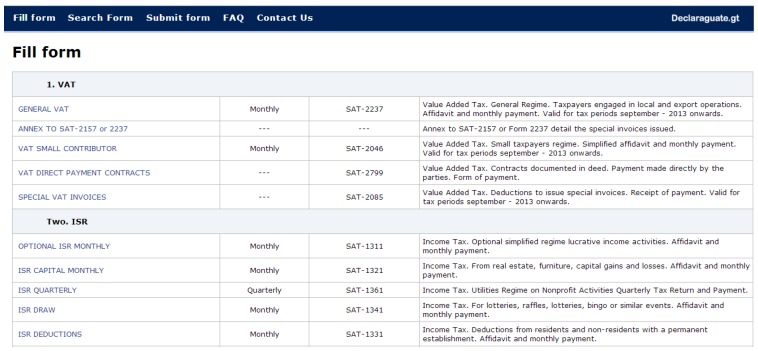
Declaraguate website home page.

**Figure A8:**
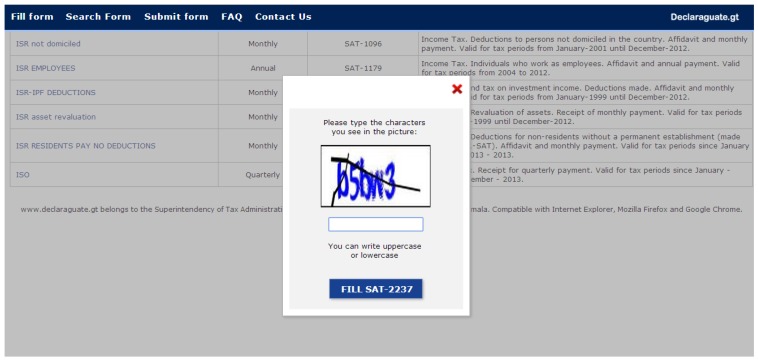
Original Captcha.

**Figure A9:**
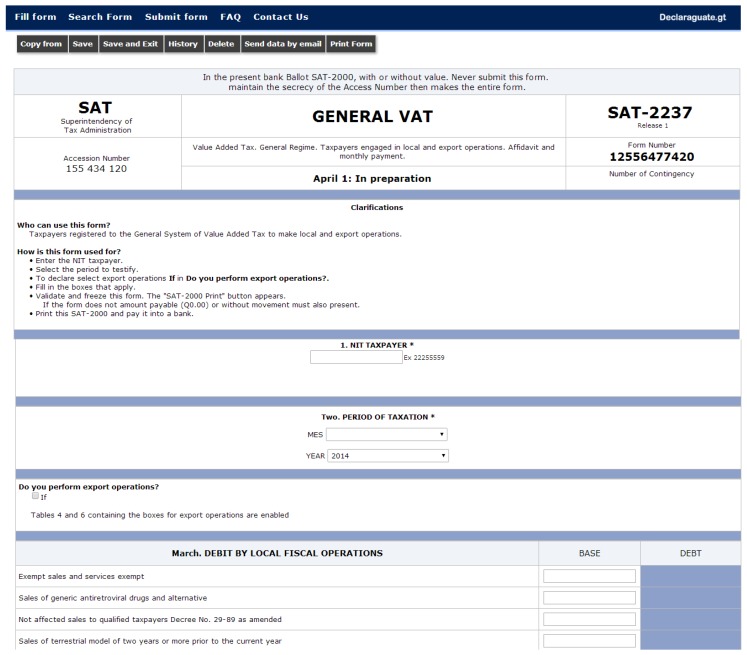
Fill Form.

This CAPTCHA allows the taxpayer to self-select into being honest. The CAPTCHA reads: ‘Which of the following do you identify with?’ The taxpayer then has to select one of the following two options: ‘I am an honest taxpayer who declares truthfully’ or ‘I am a busy taxpayer who declares quickly’. The treatment can be seen in Figure A6.

The principle applied in the Self Select ‘I am honest’ CAPTCHA is that individuals are asked to voluntarily declare their identity. The signals in our prompts are costless; thus choosing an option that signals a positive and honest indication (despite whatever declaration is actually made) should be expected of all individuals. On the one hand, this intervention might trigger an identity of an honest taxpayer [39,40,41,42], Benjamin et al. [43] find that triggering identity as a member of a religious group has significant impacts on the tendency to donate to the public good, but that this varies depending on the group identity being invoked. On the other hand, self-selection of the other (non-honesty-promoting) option is based on research that has found that people may reveal their identity (or ‘type’), even when there is no need to do so and that self-identification is not beneficial [44].

People are asked which of the two categories they would put themselves in—one is clearly positive while the other is not overly negative. The latter category is more likely to be chosen by people who would want an excuse for not filing their taxes properly and would not easily be moved by a nudge to act honestly [45]. However, everyone else will choose the former category—and they not only self-select into this category, but are being nudged into being honest (like in the ‘honesty declaration’ CAPTCHA above).

Conversely, however, the potential for ‘adverse nudging’ could apply to those who choose the second category: they might feel more justified in making a dishonest declaration. This might open the door for increased fraudulent behaviour, but might equally be offset by more targeted audits of those who self-select into the second category. It enables law enforcement to more easily identify people who might not have filed their taxes completely honestly as data will be gathered on those that self-categorised as those with too little time to do their taxes correctly to avoid the ‘honesty’ nudge.

The specific writing of this design also involves a further insight. Recently, evidence has emerged that subtle linguistic cues can have a substantial impact on our maintenance of our self-image and, consequently, on our ethical decision-making. In a study by Bryan et al. [46], the authors found that the phrase ‘I am a voter’ is more effective than ‘voting’ in inducing voter turnout because the first one feels to people like an association of their self-image (not just an act they do).

Similarly, Bryan et al. [47] find that people identify themselves with ‘I am’ more than non-identity invoked primes in the context of cheating. In three experiments people were asked not to cheat on a task. In the instructions cheating was either referred to in the context of an actor’s identity (‘Please don’t be a cheater’) or using language that referred to the action (‘Please don’t cheat’). People who had been asked not to be a cheater were less likely to cheat compared to those where the action of cheating was mentioned.

For this treatment, we hypothesise a similar cognitive dissonance to that described in the previous treatment. In addition, we hypothesise that participants will be primed with a sense of identity as an ‘honest’ individual, to which they will feel the desire to conform.

### 2.4. Outcome Variables

This trial was pre-registered in the American Economic Association’s Trial Registry (#AEARCTR-0000424) in advance of trial execution. The publicly available trial protocol included the main analysis plan, including the primary outcome variables, the regression model, and power calculations for sample size estimation based on our estimates of how the population might respond to the interventions.

The objective of this trial can be framed in one of two ways: to increase tax revenues in Guatemala, or to encourage more honesty in the declaration of tax liabilities. The first outcome variable is a continuous non-negative number of tax declared, while the latter is a binary variable that is 1 if a citizen declared any non-zero tax, and 0 otherwise. Total tax liability declared is our primary outcome measure, followed by any tax declared in our secondary analysis.

Following Shu et al. [25], we assume that if a participant is reporting their tax liability dishonestly (as opposed to making an error), they will systematically understate their liability relative to the truth. We further assume that misreporting through error is statistically similar to classical measurement error, and so is randomly distributed around the true value according to some unknown distribution. We assume that this error is (a) classical (b) orthogonal on treatment, and (c) does not interact with treatment—i.e. that the measurement error does not affect treatment. If (a) does not hold, we may experience a loss of power, and any treatment effect detected would be a lower bound on the true effect size; (b) holds by design of the trial, and the validity of (c) is irrelevant if (b) holds.

### 2.5. Sample Size Estimation

Based on previous research [12,17] we estimated the possible effect of our interventions to be around a 2% increase in total tax liability declared. We estimated the sample standard deviation to be 369.87 Quetzales (US $48.28) and based our sample size calculations on 80% power. Given these estimates, we arrived at a total sample size of 60,087 participants per arm, for a total minimum sample size requirement of 420,000 participants, although our ultimate sample size was larger. We are powered to detect effects of between 1 and 2% increases in tax declared (the sample size requirement for 1% is 241,000 per arm).

## 3. Results

### 3.1. Estimation

The outcome measure for this trial is the total tax liability declared by individuals. Although the outcome measure is relatively straightforward, the structure of the trial and data present potential complications, which we seek to address here.

As described above, participants taking part in the trial will be subject to at least one of four different forms of tax. The rates and levels of these taxes are different, and so, as discussed above, we may see differential impacts of our treatments by tax type. As such, the effect of pooled analysis across all four tax types is likely to be statistically inefficient, as the increased variance due to pooling is likely to be more detrimental to statistical power than the increased number of observations is beneficial. Hence, for each model specified below, we produce four results, one for each type of tax in our trial. Primary analysis is conducted only on ‘clean’ observations—that is, where the observation is the first time an individual has received any treatment. Primary use of all four months’ data risks underestimation or overestimation of treatment effects due to repeated exposure and ordering effects. Given the dynamic structure of our data, we remain confident that conservative effect sizes may still be detected.

Our primary model of interest is:

(1) $$Y_{\mathrm{it}}=\alpha \;+\;\beta_{1}\;+\;Y_{\mathrm{it}-x}\;+\;\beta_{2}C_{it}\;+\;\beta_{3}X_{i}\;+\;\beta_{4}t\;+\;\beta_{5}d_{it}\;+\;u_{i}$$

Where $Y_{\mathrm{it}}$ is our outcome measure, the amount of tax declared by individual i in month t; α is a constant term, capturing the amount of tax declared by participants in the control group in the first month contained in the data; and $Y_{\mathrm{it}-x}$ is a vector of lagged values of the outcome measure. Where participants have failed to make declarations in a given past period, these are set to 0. $C_{it}$ is a vector of binary treatment variables, one for each of our treatment CAPTCHAs (i.e., excluding the control CAPTCHA), set to 1 if a participant sees that CAPTCHA and 0 otherwise; $X_{i}$ is a vector of time-invariant participant characteristics, including age, gender and region (see Appendix C (Table A1, Table A2, Table A3)) for the balance check of the control variables); t is a linear time trend in trial-months; $d_{it}$ is the day within-month that the declaration is received; and $u_{i}$ is the error term.

### 3.2. Primary Analysis—First Exposure Only

Table 3, columns 1 to 5 show the parameter estimates for the Intention-to-Treat (ITT) model described above. The results show no significant impact of any of our treatments on the amount of tax declared by individuals. For reference, the mean level of the outcome measure in the control group for all participants is included at the bottom of the table. Thus, using the model specified in our trial protocol, we find no impact of any the treatment conditions. All of the secondary analysis below falls outside of our original protocol and so results are treated accordingly.

### 3.3. Secondary Analysis—Full Sample

Despite our expectation that people filing different tax types would respond differently, we do not see any differences across tax types. Thus, in Table 3 column 5, we pool tax types in order to see if increasing our power allows us to detect any effects. The results show that the parameter estimates all remain insignificant other than public good choice which becomes negative at the five percent significance level. This result is unlikely to be a robust effect as we now have 30 treatment condition parameter estimates, making a false positive likely.

Next we conduct further analysis using the full dataset available to us. That is, instead of looking at first exposures to the treatment conditions only, we expand our analysis to all interactions of individuals with the treatment conditions. This gives us over 400,000 observations for three out of the four tax types and over 3 million observations when we pool tax types. Table 4 shows the results of this analysis. The results again show no impact of any of our treatments on tax declarations. We therefore have strong evidence that none of our interventions have had an impact on the amount of tax declared by individuals. It is perhaps worth noting, however, that in our most highly powered analysis (column 5 of Table 4), which makes use of all observations of all tax types, the most effective treatment, signing first, is statistically significant at the 10% level in a two-tailed test (5% in a one-tailed test), although in samples of this size it is far from conclusive.

### 3.4. Impact of Treatments on Propensity to Declare

Finally we consider that our treatments may have an impact on the propensity to declare at all. We therefore run regressions with a binary variable as our outcome variable. Similar to our primary results, we find no impact of any of our treatments on the propensity to declare for each individual tax type. Table 5 shows the coefficients of our treatments on the propensity to declare with our taxes pooled, suggesting there was no impact on the propensity to declare. The only exception is that ‘enforcement choice’ during first exposure only has a negative impact of 0.5 percent on the propensity to declare. The combination of the relatively small effect size and the number of statistical comparisons necessary in this trial make it likely that this is a spurious effect.

## 4. Discussion

The results of this large-scale field experiment show that none of our treatments in this context had a significant impact on tax declaration. Interventions that have been shown to be successful elsewhere are found to have no impact on the amount declared in this context. Given the previous successful replication of various honesty declarations in a different field context (e.g., [26,48]), it is important to consider what contextual factors could have caused this failure to replicate.

We speculate that this could be for a number of reasons. First, the interventions were not a part of the form themselves (as, for example, they were in Shu et al. [25]). As we were not able in this trial to alter the forms themselves, the interventions were placed in a pop-up window as part of a CAPTCHA. This could have meant that the messages were too far separated from the filling in of the tax declaration, which appears to be in keeping with the findings of Bhanot [26]. Second, the setting of the CAPTCHA itself could have negated the impact of the messages, as CAPTCHA participants may simply ignore the extraneous prompts in a bid to progress to the main form. This could have made our treatments seem like a task rather than inviting thought about their content. The fact that all six treatments were found to be ineffective (rather than some) supports the hypothesis that the setting in which the information was conveyed was crucial here, rather than the content of the messages. Third, online nudges in some settings, including honesty primes with e-signatures, have been shown to be ineffective in prompting honesty [49]. Fourth, the Guatemalans who choose to declare their tax might do so honestly. In Guatemala there are limited consequences for not paying your tax; failure to declare is highly unlikely to result in prosecution. Thus, the taxpayers who do declare, and are thus part of our sample, may therefore be the subset of Guatemalans who do declare their taxes more honestly than the rest of the population that we were not affecting with this online intervention. Fifth, the intervention designs might simply not have had an impact on honesty in this context. Further research will be needed to investigate to what extent and under what circumstances the interventions studied here do or do not promote honest tax declarations.

It is important to place this result in the context of the wider and growing behavioural science literature around tax compliance. Much of this literature is concerned with either binary payment/declaration decisions, or the timeliness of those decisions (for example Hallsworth et al. [50]), or with honesty measured after an audit. Our paper differs from these for two reasons. First, as stated above, the auditing regime in Guatemala, as well as the consequences for non-compliance, are very weak, and so follow-up to measure truth-telling is not possible. Second, the context of this study means that it takes place ‘downstream’ of papers such as Hallsworth et al. [50], and the binary compliance decision has already taken place. We therefore consider our interventions to be an application of the findings of Shu et al. [25] who in their field experiment consider the decision whether or not to lie on car insurance forms, and therefore arguably more related to the substantive literature on honesty in behavioural science than to the tax compliance literature. As with Shu et al. [25], the (lack of a) monitoring regime requires the assumption that people systematically lie in a direction that aligns with their financial incentive not to pay tax, but we believe this assumption not to be onerous.

## 5. Conclusions

Our null results have important implications for both academic researchers and practitioners. While previous research on tax compliance [13,18,50] has shown that messaging and framing around tax choices can be an effective means of promoting more honesty in tax declarations, our experiment demonstrates that previous findings cannot simply be applied in any context. Differences between countries, institutions, and settings matter and require careful consideration before similar interventions are scaled up in a new context. For instance, our setting differs notably from previous studies in that the interventions to increase tax compliance were delivered online, not in a letter as in past research. Work by Chou [49] suggests one potential reason for the failure of our sign-before intervention: signing one’s name digitally does not have the same symbolic meaning to signers and consequently does not invoke one’s identity in the same way. More research is required to understand what, and *how*, interventions work (or occasionally fail to work) in real policy contexts. It also highlights the importance of testing before implementing a new policy idea, providing more evidence for the need for evidence-based policy-making [5].

Finally, publishing null results is becoming increasingly more common in psychology, economics, policy and other disciplines. Doing so helps reduce the file-drawer problem [51] and adds to our understanding of the tested interventions and their boundary conditions [52]. All in all, this helps evidence-based policy-makers make more informed decisions.

## Figures and Tables

**Table 1:** Descriptive statistics of our sample (mean and standard deviation). Column 1 shows the proportion of taxpayers who are women; column 2 lists the mean age in years; and columns 3–6 show the proportion of taxpayers in each of the four tax types.

Condition	Female	Age	Tax Type Filed (Proportion Declaring)			
			Gross Income	Profits Income	VAT Small Taxpayers	VAT General
Control Group	0.375	37.34	0.156	0.0202	0.526	0.297
	(0.484)	(17.91)	(0.363)	(0.141)	(0.499)	(0.457)
Honesty Declaration	0.374	37.47	0.154	0.0193	0.532	0.294
	(0.484)	(17.80)	(0.361)	(0.137)	(0.499)	(0.456)
Public Good	0.375	37.32	0.157	0.0204	0.528	0.295
	(0.484)	(17.93)	(0.364)	(0.141)	(0.499)	(0.456)
Enforcement	0.375	37.26	0.157	0.0201	0.528	0.295
	(0.484)	(17.93)	(0.364)	(0.140)	(0.499)	(0.456)
Choice Public Good	0.374	37.30	0.159	0.0206	0.524	0.296
	(0.484)	(17.96)	(0.366)	(0.142)	(0.499)	(0.456)
Choice Enforcement	0.372	37.34	0.161	0.0204	0.522	0.297
	(0.483)	(17.97)	(0.367)	(0.141)	(0.500)	(0.457)
Self-select, ‘I am Honest’	0.374	37.32	0.159	0.0199	0.527	0.295
	(0.484)	(17.93)	(0.365)	(0.140)	(0.499)	(0.456)
Total	0.374	37.34	0.158	0.0201	0.527	0.296
	(0.484)	(17.92)	(0.364)	(0.140)	(0.499)	(0.456)

**Table 2:** Summary of treatment arms and behavioural prompts. Number of forms submitted (***n***) is cumulative over the entire trial period.

Condition	Message and Procedure
Control Group, *Number of forms submitted = 585,872*	■ Typical CAPTCHA website design. Box states ‘please type the characters that you see in the picture’. ■ Once the characters from the picture are typed, the taxpayer is then able to click on the ‘fill form’ button to take them to the declaration form.
Honesty Declaration, *Number of forms submitted = 529,397*	■ Includes an honesty declaration that translates as: ‘Declaration: I will fill out this form honestly. Please sign your name to confirm this declaration’ ■ The taxpayer must then enter their name in a box below this statement before being able to press the ‘fill form’ button.
Public Good, *Number of forms submitted = 573,676*	■ Includes an image of the Guatemalan flag, and the following public good message: ‘In 2013 your taxes helped pay for schools, hospitals and policemen.’
Enforcement, *Number of forms submitted = 563,670*	■ Includes an image of a gavel and the following text: ‘5,060 taxpayers in early 2014 had legal proceedings for breach of their tax obligations.’
Choice Public Good, *Number of forms submitted = 548,915*	■ Gives the taxpayer a choice of public goods that they would like to see tax money spent on: ‘Please choose what you want us to direct your tax money to:’ ■ The taxpayer must choose one of the options: schools; hospitals; or policemen, before they are able to press the ‘fill form’ button.
Choice Enforcement, *Number of forms submitted = 539,542*	■ Gives the taxpayer a choice of the punishment that they think people should receive for fraudulently declaring their tax: ‘Please tell us what you think should happen to people who fill out their forms dishonestly’ ■ The taxpayer must choose one of the options: pay a fine; confiscate your assets; or go to jail, before they are able to press the ‘fill form’ button.
Self Select ‘I am Honest’, *Number of forms submitted = 545,168*	■ Allows the taxpayer to self-select into being honest: ‘Which of the following do you identify with?’ ■ The taxpayer then has to select one of the following two options: ‘I am an honest taxpayer who declares truthfully’ or ‘I'm a busy taxpayer who declares quickly’

**Table 3:** Linear regression ITT estimates of treatment impacts on tax declaration (in Quetzales) with the control condition as baseline. First exposure to any treatment only. Robust standard errors in parentheses.

Variable	(1)	(2)	(3)	(4)	(5)
	(Gross Income)	(Profits Income)	(VAT Small)	(VAT General)	(Pooled Taxes)
Sign before	−6.432	−71.332	−0.560	−2.468	−1.170
	(−12.468)	(−60.643)	(−0.643)	(−4.644)	(−1.273)
Public Good	10.244	−86.205	−0.291	−9.302	−1.200
	(−20.846)	(−66.939)	(−0.615)	(−8.405)	(−2.080)
Enforcement	2.094	−64.222	−0.397	−3.472	−1.639
	(−13.704)	(−74.212)	(−0.658)	(−4.774)	(−1.263)
Public Good Choice	−0.783	−89.340	−0.772	−8.419	−2.603 *
	(−11.658)	(−62.236)	(−0.661)	(−5.100)	(−1.315)
Enforcement Choice	−7.950	−102.452	−0.019	1.178	−0.580
	(−12.840)	(−67.784)	(−0.685)	(−10.492)	(−2.370)
Self-select Honesty	20.135	−46.944	−0.971	−1.382	0.204
	(−15.040)	(−65.386)	(−0.613)	(−5.341)	(−1.577)
Age	−0.426	−0.605	−0.030 *	−0.219	−0.142 *
	(−0.281)	(−0.745)	(−0.014)	(−0.154)	(−0.058)
Female	−20.820 **	39.438	−0.321	−1.231	−0.667
	(−7.546)	(−36.834)	(−0.374)	(−2.805)	(−0.793)
Central Region	21.652	−4.330	7.092 ***	7.296	5.533 ***
	(−11.091)	(−15.456)	(−0.513)	(−4.109)	(−1.064)
Northern Region	22.997	89.048	−1.528 *	−0.428	0.149
	(−15.765)	(−45.614)	(−0.609)	(-6.604)	(−1.489)
Eastern Region	−2.401	45.397	1.735 ***	7.947	2.885 *
	(−14.240)	(−45.505)	(−0.504)	(−5.353)	(−1.149)
Access Month	29.559 *	147.802 *	20.001 ***	22.070 ***	22.717 ***
	(−14.181)	(−66.204)	(−0.514)	(−6.069)	(−1.086)
Access Day	3.527 **	7.665 *	0.803 ***	0.657 **	0.848 ***
	(−1.185)	(−3.422)	(−0.032)	(−0.201)	(−0.038)
Constant	−71.210	−278.628 *	−40.138 ***	−40.981 *	−43.749 ***
	(−43.955)	(−127.002)	(−1.663)	(−18.079)	(−3.038)
Lagged Declarations	Yes	Yes	Yes	Yes	Yes
Control Mean	190.27	90.73	58.70	270.47	109.18
Observations	24641	2177	467409	133015	627242

* *p* < 0.05, ** *p* < 0.01, *** *p* < 0.001.

**Table 4:** Linear regression ITT estimates of treatment impacts on tax declaration (in Quetzales) with the control condition as baseline and control variables. Full sample of all tax declarations. Robust standard errors clustered at the individual level in parentheses.

Variable	(1)	(2)	(3)	(4)	(5)
	(Gross Income)	(Profits Income)	(VAT Small)	(VAT General)	(Pooled Taxes)
Sign before	6.224	81.742	−0.286	19.266	6.598
	(7.483)	(105.072)	(0.389)	(15.302)	(3.861)
Public Good	1.192	−41.440	−0.512	−4.228	−1.152
	(6.234)	(50.409)	(0.372)	(12.568)	(3.089)
Enforcement	0.563	−4.401	−0.271	8.405	1.765
	(6.592)	(44.509)	(0.383)	(14.419)	(3.491)
Public Good Choice	−2.609	−52.511	−0.345	9.547	2.162
	(5.887)	(41.748)	(0.387)	(14.896)	(3.558)
Enforcement Choice	−7.217	−35.598	0.641	3.806	0.300
	(6.313)	(67.750)	(0.425)	(14.026)	(3.398)
Self-select Honesty	0.162	193.019	−0.191	−12.334	-0.536
	(5.992)	(164.711)	(0.385)	(12.415)	(3.353)
Age	−0.350 *	−3.387	−0.009	0.729*	0.396 **
	(0.163)	(2.352)	(0.010)	(0.340)	(0.132)
Female	−14.913 ***	6.568	−0.557 *	−40.296 ***	−14.092 ***
	(4.028)	(55.787)	(0.241)	(6.401)	(1.954)
Central Region	16.549 *	50.940	8.273 ***	74.527 ***	33.112 ***
	(8.239)	(40.881)	(0.301)	(10.841)	(3.646)
Northern Region	−0.525	−46.185	−4.282 ***	33.353 **	4.664
	(11.834)	(30.883)	(0.312)	(12.848)	(2.605)
Eastern Region	3.838	−31.319	−0.431	33.614 ***	5.369 **
	(9.135)	(25.455)	(0.317)	(9.441)	(1.886)
Access Month	3.989 **	90.629	0.800 ***	−4.263	−0.673
	(1.509)	(59.315)	(0.083)	(2.367)	(0.601)
Access Day	2.677 ***	3.163	0.082 ***	0.746 *	0.120
	(0.631)	(3.328)	(0.012)	(0.376)	(0.103)
Constant	−6.622	−126.860	10.202 ***	−6.060	5.706
	(12.560)	(131.249)	(0.834)	(19.377)	(5.276)
Lagged Declarations	Yes	Yes	Yes	Yes	Yes
Control Mean	259.07	207.88	58.18	419.32	169.07
Observations	423832	26983	2036259	745356	3232430

* *p* < 0.05, ** *p* < 0.01, *** *p* < 0.001.

**Table 5:** Treatment impacts on propensity to declare a non-zero amount; all tax types are pooled.

Variable	(1)	(2)
	First Exposure	Full Sample
Sign before	0.002	0.001
	(0.002)	(0.001)
Public Good	−0.001	−0.001
	(0.002)	(0.001)
Enforcement	0.000	0.001
	(0.002)	(0.001)
Public Good Choice	−0.002	−0.001
	(0.002)	(0.001)
Enforcement Choice	−0.005 *	−0.002
	(0.002)	(0.001)
Self-select Honesty	−0.001	-0.000
	(0.002)	(0.001)
Age	0.002 ***	0.002 ***
	(0.000)	(0.000)
Female	0.039 ***	0.031 ***
	(0.001)	(0.001)
Central Region	−0.061 ***	−0.064 ***
	(0.002)	(0.002)
Northern Region	−0.041***	−0.035 ***
	(0.002)	(0.002)
Eastern Region	−0.054 ***	−0.047 ***
	(0.002)	(0.002)
Trial Month	0.031 ***	−0.001 ***
	(0.001)	(0.000)
Access Day	−0.000	0.000 ***
	(0.000)	(0.000)
Constant	0.255 ***	0.334 ***
	(0.004)	(0.003)
Lagged Declarations	Yes	Yes
Observations	627242	3232430

*p < 0.05, ** *p* < 0.01, *** *p* < 0.001.

## Appendix C. Balance Checks

The primary assumption of randomisation is that it creates balance on both observable and unobservable covariates. Given that randomisation was conducted by an automated process, in real time, on the tax website of Guatemala, in this appendix we consider basic analysis of the balance of covariates prior to the experiment.

The tables below estimate a simple regression model, in which the baseline (prior to the experiment) of tax payments, and age and gender in our analysis is regressed in turn on the full set of binary treatment variables. In each table, column 1 assesses these variables for the first time an individual is treated, while column 2 considers it for the full sample of observations.

**Table A1:** Balance of treatments with respect to age.

Variable	(1)	(2)
	(First)	(All)
Sign before	0.080	0.130 ***
	(0.074)	(0.034)
Public Good	0.044	−0.015
	(0.073)	(0.033)
Enforcement	0.018	−0.077 *
	(0.073)	(0.033)
Sign before	0.045	−0.033
	(0.074)	(0.034)
Public Good	0.004	0.004
	(0.075)	(0.034)
Enforcement	0.034	−0.016
	(0.074)	(0.034)
Constant	37.906 ***	37.338 ***
	(0.051)	(0.023)
Observations	715,026	3,885,225

* *p* < 0.05, ** *p* < 0.01, *** *p* < 0.001.

**Table A2:** Balance of treatments with respect to proportion female.

Variable	(1)	(2)
	(First)	(All)
Sign before	−0.005 *	−0.001
	(0.002)	(0.001)
Public Good	−0.002	0.000
	(0.002)	(0.001)
Enforcement	0.002	0.001
	(0.002)	(0.001)
Sign before	−0.000	−0.000
	(0.002)	(0.001)
Public Good	−0.004	−0.002 *
	(0.002)	(0.001)
Enforcement	−0.002	−0.000
	(0.002)	(0.001)
Constant	0.384 ***	0.375 ***
	(0.002)	(0.001)
Observations	627,300	3,232,686

**p* < 0.05, ***p* < 0.01, *** *p* < 0.001.

**Table A3:** Balance of treatments with respect to prior tax declarations.

Variable	(1)	(2)
	(First)	(All)
Sign before	−164.272	−1188.269
	(2263.356)	(1264.250)
Public Good	−856.039	692.126
	(2213.478)	(1238.355)
Enforcement	−605.685	−1118.926
	(2230.887)	(1243.896)
Sign before	2424.769	38.043
	(2261.094)	(1252.387)
Public Good	−1654.504	147.031
	(2277.990)	(1257.991)
Enforcement	−3296.176	−1779.398
	(2266.044)	(1254.607)
Constant	10,422.377 ***	12,294.340 ***
	(1551.063)	(871.032)
Observations	715,190	3,886,240

* *p* < 0.05, ** *p* < 0.01, *** *p* < 0.001.
